# RNA-Seq analysis uncovers transcriptomic variations between morphologically similar in vivo- and in vitro-derived bovine blastocysts

**DOI:** 10.1186/1471-2164-13-118

**Published:** 2012-03-28

**Authors:** Ashley M Driver, Francisco Peñagaricano, Wen Huang, Khawaja R Ahmad, Katie S Hackbart, Milo C Wiltbank, Hasan Khatib

**Affiliations:** 1 Department of Dairy Science, University of Wisconsin, Madison, WI 53706, USA; 2 Department of Animal Sciences, University of Wisconsin, Madison, WI 53706, USA; 3 Department of Biological Sciences, University of Sargodha, Sargodha, Pakistan

**Keywords:** In vivo embryo, In vitro fertilization, RNA-Seq, Transcriptome, Alternative splicing

## Abstract

**Background:**

A valuable tool for both research and industry, in vitro fertilization (IVF) has applications range from gamete selection and preservation of traits to cloning. Although IVF has achieved worldwide use, with approximately 339,685 bovine embryos transferred in 2010 alone, there are still continuing difficulties with efficiency. It is rare to have more than 40% of fertilized in vitro cattle oocytes reach blastocyst stage by day 8 of culture, and pregnancy rates are reported as less than 45% for in vitro produced embryos. To investigate potential influences in-vitro fertilization (IVF) has on embryonic development, this study compares in vivo- and in vitro-derived bovine blastocysts at a similar stage and quality grade (expanded, excellent quality) to determine the degree of transcriptomic variation beyond morphology using RNA-Seq.

**Results:**

A total of 26,906,451 and 38,184,547 fragments were sequenced for in vitro and in vivo embryo pools, respectively. We detected expression for a total of 17,634 genes, with 793 genes showing differential expression between the two embryo populations with false discovery rate (FDR) < 0.05. There were also 395 novel transcribed units found, of which 45 were differentially expressed (FDR < 0.05). In addition, 4,800 genes showed evidence of alternative splicing, with 873 genes displaying differential alternative splicing between the two pools (FDR < 0.05). Using GO enrichment analysis, multiple biological pathways were found to be significantly enriched for differentially expressed genes (FDR < 0.01), including cholesterol and sterol synthesis, system development, and cell differentiation.

**Conclusions:**

Thus, our results support that IVF may influence at the transcriptomic level and that morphology is limited in full characterization of bovine preimplantation embryos.

## Background

The use of in vitro fertilization (IVF) in cattle and in other species such as humans has seen rapid increases in the last few decades. In 2010 alone, over 339,685 in vitro-produced bovine embryos were transferred worldwide [1]. In addition 60,190 infants were born in the United States in 2009 from assisted reproductive technologies (ART), 99% of which involved IVF [2]. In addition, the International Committee for Monitoring Assisted Reproductive Technology's (ICMART) estimated that in 2003, approximately 232,000 babies had been born by ART worldwide [3].

Although the statistics reflect a striking amount of IVF use, they also depict relatively low success rates. It is rare to have more than 40% of fertilized in vitro cattle oocytes reach blastocyst stage, and of those transferred pregnancy rates are less than 45% for in vitro techniques [4]. Similarly in humans, statistics from 2007 show an approximate 32-37% live birth rate from transfers [2]. As such, there is increasing pressure to better understand the embryo's biological framework in order to interpret the seemingly low developmental rates. The reported sensitivity of the embryo to environmental factors, such as pH and ionic stress during the period in which they are manipulated for in-vitro procedures has raised further importance for more in-depth studies [5].

Morphologically, distinction between in vivo and IVF pre-implantation embryos have been confirmed by characteristics such as differing cytoplasmic color [6], lipid droplet composition [7], and mitochondrial content [8]. A comparison of in vitro and in vivo blastocysts via light microscopy showed lower microvilli coverage and lipid content in the in vitro-produced embryos [9]. In addition to morphology, previous studies have ascertained differences metabolically [10] and at the chromosomal level, with in vitro embryos incurring higher incidences of abnormalities such as mixiploidy [11].

Differential gene expression has gained increasing attention as a mechanism underlying the phenotypic abnormalities between in vivo and in vitro embryos [9,12-15]. In addition to cattle, these differences have also been reported in sheep [16] and mice [17], suggesting that the IVF process itself may influence the biological framework of the early developing embryo. These studies, however, utilized expression microarrays, which provide limited transcriptomic data. In addition, in vivo embryos were derived from a limited number of super-ovulated donor cows, which presents a confounding variable of potential superovulation effects. In contrast, this study utilizes in vivo embryos from non-superovulated cows to eliminate potential sources of transcriptomic influence to characterize transcriptomic variations due to the in vitro process itself. We hypothesize that in vivo and in vitro embryo populations differ in their biological and transcriptomic characteristics and that the identification of these differences can assist in understanding the molecular mechanisms influencing development of IVF embryos. This study reports a fine scale assessment of the differential gene expression and alternative splicing between bovine in vivo- and in vitro-derived embryos to provide potential transcriptomic characteristics for further investigation.

## Results

### Deep sequencing of the bovine blastocyst transcriptomes

Using RNA-Seq, this study was able to characterize the transcriptomic landscapes of in vivo- versus in vitro-derived bovine blastocysts. In order to accomplish this, two rounds of linear amplification of mRNA were used to ensure that individual embryos produced enough RNA input for analysis. Amplified RNA from five individual in vitro-derived and five in vivo-derived embryos all with the same sire was pooled, multiplexed, and sequenced on the HiSeq2000, producing approximately 60 million pair-end reads of 100 bp in length. Table 1 displays the overall results of sequencing read alignments to the bovine reference genome.

**Table 1:** Summary of sequence read alignments to the reference genome

Sample	In vitro embryos	In vivo embryos
Pair end reads	26,906,451 x 2	38,184,547 *x*2

Total sequenced fragments	26,906,451	38,184,547

Total mapped fragments	22,428,488	32,384,577

Uniquely mapped fragments	20,389,330	29,728,363

Fragments mapped to autosomes and X chromosome	19,556,926	28,362,794

Fragments mapped to annotated genes	13,304,981	16,976,922

Fragments mapped to annotated exons	6,942,761	8,185,094

Fragments overlapped with annotated introns	6,362,220	8,791,828

Analysis of sequencing reads was done using Tophat software [18] for alignment with the reference genome (btau4.0). Of the total sequenced fragments, 83% and 85% were mapped to the reference genome for in vitro and in vivo embryos, respectively, and of these, 91% and 92%, respectively, were uniquely mapped to specific regions in the bovine genome (Table 1). Of those uniquely mapped, 19,556,926 and 28,326,794 were mapped to one of the autosomes or X chromosome for in vitro and in vivo embryos, respectively (Table 1).

### Identification and analysis of novel transcribed units

Using stringent criteria, our analysis uncovered a total of 395 novel transcribed units. Of these, 45 show differential expression (FDR < 0.05), with 23 transcripts over-expressed in the in vivo embryos and 22 over-expressed in the in vitro embryos. Three transcripts had exclusive expression in the in vivo population while one transcript was exclusive to in vitro-produced embryos. When compared to known cattle ESTs from the UCSC genome browser [19], we found that 237 novel transcripts were supported by at least five cow ESTs with total coverage greater than 50%. Twenty of these transcripts showed significant differential expression.

### Identification and validation of differentially expressed genes

The RNA-Seq technique allows analysis of the differential expression profile via transcript abundance with a high sensitivity for lowly expressed transcripts that would otherwise be undetected by standard microarrays [20]. Overall we tested 17,634 genes for expression analysis, with 793 genes showing differential expression between in vitro and in vivo embryos (FDR < 0.05). Of these, 37 genes were exclusively expressed in the in vivo population and 10 genes were exclusively expressed in the in vitro population. Of the remaining 746 genes, 549 showed higher expression in the in vivo embryos versus 197 genes that showed higher expression in the in vitro embryos. Table 2 shows the breakdown of fold-change among the differentially expressed genes between the two populations.

**Table 2:** Fold change differences in expression of genes differentially expressed between in vivo and in vitro embryos at FDR < 0.05

	Fold change				
	
	≤ 5	5-10	10-15	15-20	> 20
Genes over-expressed in in vivo embryos	132	108	93	55	161

Genes over-expressed in in vitro embryos	51	42	34	19	51

**Figure 1:**
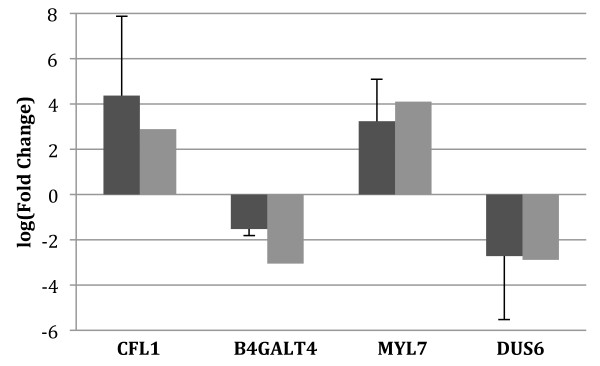
**Comparison of changes in four differentially expressed genes from the RNA-Seq results (light grey) vs. qRT-PCR (dark grey)**. Bars above the X-axis denote genes with higher expression in the in vitro embryos while bars below the X-axis denote genes with higher expression in the in vivo embryos.

For the validation of RNA-Seq results, four genes showing high levels of significance (*CFL1, MYL7, DUS6*, and *B4GALT4*) were selected and normalized to *GBG5 *via qRT-PCR in the individual embryo aRNA samples. Two genes, *CFL1 *and *MYL7*, showed higher expression in the in vitro embryos with an average of 79.5- and 25.5-fold differences, respectively, when measured with qRT-PCR and 18- and 60-fold differences, respectively, using RNA-Seq (Figure 1). The other two genes, *DUS6 *and *B4GALT4*, showed higher expression in the in vivo embryos, with an average of 15- and 4.6-fold differences, respectively using qRT-PCR and 21- and 18-fold difference, respectively, with RNA-Seq (Figure 1). Thus, these four genes showed similar patterns of mRNA abundance in RNA-Seq analysis and qRT-PCR.

### Differential alternative splicing between in vivo and in vitro embryos

In all, we detected a total of 4,800 genes expressing more than one splice variant. For further analysis, we used genes that had multiple isoforms and had a single transcription start site (n = 2,457 genes). Of these, 873 showed differential alternative splicing between the two embryo populations (FDR < 0.05). Moreover, following Wang et al. [21], 2,329 isoforms from 1,493 genes were classified into a total of 2,778 simple alternative events that fell into six different categories (Table 3). It is important to remark that some genes showed more than one variable splicing event. The rest of the genes (n = 964) showed more complicated alternative splicing events and it was not possible to classify the events into these simple six categories.

**Table 3:** Classification of genes with simple alternative splicing events or differential alternative splicing

Alternative splicing event	No. of events	No. of genes	No. of genes that showed differential splicing*
Exon skipping	1147	684	276

Alternative 5'	551	344	142

Alternative 3'	353	265	121

Intron retention	404	264	106

Mutually exclusive	62	53	21

Alternative last exon	261	181	66

*Differential alternative splicing between in vivo- and in vitro-derived embryos

**Figure 2:**
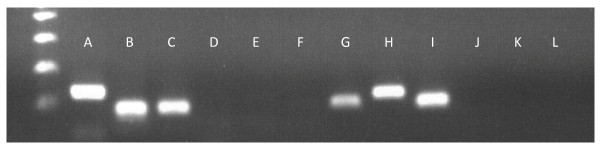
**Alternative splicing validations for two genes using PCR with cDNA as the template**. Lanes A and B correspond to the two fragment sizes for the gene *AP2B1 *(134 and 92 bp) while G and H represent the two transcripts for *ZDHHC16 *(97 and 118 bp). Lanes C and I are positive controls (*GBG5*, 115 bp) while D, E, F, J, K, and L are negative controls.

Validation of alternative splicing was confirmed for the genes *AP2B1 *and *ZDHHC16*. Both of these genes showed evidence for single exon skipping. As such, primers were designed around this region to confirm the splicing event. The first primer set for *AP2B1 *had exon inclusion and produced a 134-bp product in comparison to the second primer set excluding the exon which resulted in a 92-bp product (Figure 2). For *ZDHHC16*, two primers sets were also created, with the first set producing a 97-bp product (transcript that excludes the exon) and the second set producing a 118-bp product that included the skipped exon (Figure 2). Since both transcripts were present in both embryo samples for each gene, although differing in ratio, they were tested in a pool each of in vivo and in vitro embryo cDNA for validation. Overall, RNA-Seq results for alternative splicing were also validated.

### Gene ontology analysis

To gain insights into the biological processes that could be regulated differently between in vivo and in vitro embryos, we performed a GO analysis. We found a total of 23 pathways significantly enriched (FDR < 0.01) for differentially expressed genes, many of which are related to developmental processes (Additional file 1). The pathway showing the highest level of significance was cholesterol biosynthesis. Eleven genes involved in this pathway showed higher expression in the in vitro embryos compared to the in vivo embryos (Table 4). Furthermore, after cholesterol biosynthesis, the pathways of system development, sterol synthesis, and cell differentiation showed the highest significance in the GO analysis (Additional file 1).

**Table 4:** Fold difference and q-values of 11 cholesterol biosynthesis pathway genes

Gene ID	Gene Name	Fold change	q-value
ENSBTAG00000011839	*HMGCS1*	5.10	< 0.001

ENSBTAG00000007840	*HMGCR*	3.85	< 0.001

ENSBTAG00000017819	*PMVK*	9.51	< 0.001

ENSBTAG00000012059	*MVD*	2.62	0.047

ENSBTAG00000004075	*IDI1*	3.83	0.002

ENSBTAG00000012432	*FDFT1*	3.32	0.012

ENSBTAG00000005498	*SQLE*	3.34	0.004

ENSBTAG00000018936	*LSS*	7.75	< 0.001

ENSBTAG00000001992	*CYP51*	5.93	< 0.001

ENSBTAG00000003068	*SC4MOL*	2.53	0.036

ENSBTAG00000016465	*DHCR7*	4.68	< 0.001

All listed genes showed higher expression in the in vitro embryos

## Discussion

### Depth of RNA-Seq analysis

Our current knowledge on dynamic changes in gene expression during embryonic development has for the most part been based on results from microarray technology. Although providing insight, microarrays are based on *a priori *knowledge and oligonucleotide design and may not detect lowly expressed transcripts or provide complete genome coverage due to lack of probe availability. Also, expression microarrays do not provide information regarding alternative splicing and novel transcripts. Therefore, we utilized RNA-Seq technology in this study to perform an in-depth analysis of the transcriptomic landscape of bovine in vivo- and IVF-derived embryos at the blastocyst stage. This is the first report of a high-resolution snapshot of transcriptomic differences between in vivo and in vitro embryos beyond expression for any species using RNA-Seq technology.

Another strength of RNA-Seq technology is the ability to improve genome annotation by the discovery of novel transcripts. In our study, we identified 307,646 bp covering novel exonic regions from 395 novel transcript units. Of these, 45 transcripts showed differential expression between in vivo and in vitro embryos (FDR < 0.05), with 23 expressed higher in the in vivo embryos and 22 in the in vitro embryos. In addition, three novel transcripts were exclusive to the in vivo embryos and one to the in vitro. Thus, this is evidence that not only are there uncharacterized regions of the bovine genome, but also that these novel regions may serve important biological roles.

### Beyond morphology

Although the two embryo groups possessed similar morphological appearance and would have received a similar rating for developmental stage and embryo quality, they possessed numerous transcriptomic differences. Of the 793 genes showing differential expression, 413 had greater than a 10-fold difference in mRNA concentrations.

In addition, RNA-Seq technology provided valuable information regarding alternative and novel splice variants reflecting more complex mechanisms of RNA regulation. A recent study by Corcoran et al. [13] found that many genes involved in RNA processing and translation were differentially expressed between in vivo and in vitro blastocyst populations. Our study extends these observations by identifying a number of alternatively spliced genes between in vivo and in vitro populations.

Overall, our study detected 873 genes with alternative splicing, and of these, 782 showed differential splicing without showing overall differential gene expression. This is critical as alternative splicing can influence protein production and biological function. Associations between differential alternative splicing and embryonic development have been reported by Zhang et al. [22]. This study reported differential expression levels of alternative transcripts for heat shock proteins in developmentally arrested in vitro bovine embryos in comparison to developed blastocysts, with the suggestion that differential processing of RNA is associated with regulation of embryonic development. Thus, our study is presenting transcriptomic information that would otherwise not be detected by standard microarray and yet have key biological importance for development.

Beyond individual gene differences, our study also revealed several biological pathways that were significantly enriched or depleted based on substantial differences in embryonic mRNA concentrations. The 23 pathways showing the highest level of significance can be found in Additional file 1.

Of particular interest was the cholesterol biosynthesis pathway, which had 11 genes with increased expression in the in vitro embryos. Differential expression levels of the cholesterol biosynthesis pathway between our embryo populations could be supported by a study conducted by Tint et al. establishing that the mother provides necessary cholesterol to the in vivo embryo throughout the pre-implantation period [23]. Since in vitro embryos lack contact with maternal tissues, and thus maternal sources of cholesterol, there could be increased de novo production of cholesterol.

Of the differential genes listed in Table 4, many have been shown to be critical to the cholesterol biosynthesis process. For example, lanosterol synthase (*LSS*) catalyzes (S)-2,3 oxidosqualene into the parental compounds for cholesterol and ultimate steroid synthesis in mammals [24]. In addition, the gene *HMGCR *contributes to the major rate-limiting step for conversion of HMG-CoA into mevalonate [25]. *HMGCR *is also regulated through a feedback system, which could be altered by a lack of maternal influence and delivery on the embryo [25]. Should the proper levels of these genes be perturbed, it could have significant effects on an organism by altering products of cholesterol (i.e. steroids). Interestingly it should be noted that another pathway related to steroid production (sterol biosynthesis) was also enriched between in vivo and in vitro embryos.

In addition, the cell differentiation pathway was enriched which is intriguing as differentiation is not only critical for the blastocyst formation but to ensure proper implantation and placenta development [26]. This finding supports earlier histological studies showing fewer ICM cells in in vitro-produced bovine embryos compared to in vivo [27]. Detection of this differential pathway in our study provides further evidence for its potential importance, thus helping to further the ongoing characterization of in vivo and in vitro embryos. In conclusion, our results suggest that IVF may influence at a larger scale by altering biological pathways critical for early development.

### Potential limitations

One limitation to this study is the potential shortening of reads due to the RNA amplification process, which tends to favor the 3' end of the sequence. In addition this may cause a lack of complete information on full length alternative splicing. Due to limited amounts of RNA in individual embryos, two rounds of linear amplification were necessary. Although it remains unclear as to the degree of transcript shortening that occurs, it should be acknowledged as it may prevent construction of full length transcripts as discussed by Huang and Khatib [28]. Nonetheless, amplification provided a means to utilize individual embryos for validation and pool construction.

A second limitation to this study was the lack of RNA-Seq replicates. Nonetheless, in a prior RNA-Seq study by our lab, two pools were utilized and results were consistent with qRT-PCR validations and prior microarray results that used replicates [28]. Thus, even with limited number of samples, RNA-Seq results from this study provide valuable insight into the genomic characteristics of embryo populations. As such, although there are acknowledged limitations, using RNA-Seq approach as a hypothesis-generating study provides numerous opportunities for further investigation to better understand early embryonic development.

## Conclusions

Use of IVF and in vitro embryo culture has been found to be a valuable tool for both research and industry with applications ranging from infertility treatment, and gamete selection to cloning. In addition, the bovine embryo model has become an interesting and popular biological model to help in understanding potential problems or optimization procedures for human embryonic practices [29]. As such, results of this study may help elucidate the molecular mechanisms leading to seemingly low success rates of IVF embryos and may help to unlock important factors underlying the early embryo's growth and sensitivity during the pre-implantation period. Overall, our results support the initial hypothesis that in vitro embryos differ from their in vivo counterparts on multiple transcriptomic levels. In addition, this study highlighted the importance of studying RNA processing beyond the levels of expression and the limitations morphology alone has on embryo characterization. Candidate genes and pathways uncovered in this study can be pursued in future studies with the goal of improvement of in vitro fertilization and culture systems and to further our understanding of factors involved in optimal embryonic development.

## Methods

### In vivo embryo collection and grading

**Figure 3:**
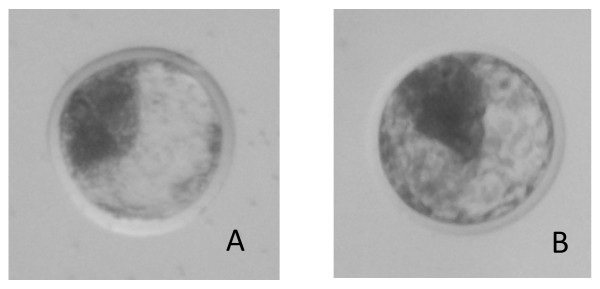
**Morphological assessment of embryos at the blastocyst stage with a grade of 7-1 according to IETS standards**. A = in vivo blastocyst; B = in vitro blastocyst.

In vivo embryos were collected from non-superovulated lactating Holstein cows (n = 84, multiparous; approximately 77 days after calving) at the University of Wisconsin-Madison. To allow a synchronous follicular development and tight regulation of ovulatory follicle size and time of ovulation, cows were synchronized with the Double OvSynch protocol similar to that described in Souza et al. [30]. Most cows have single ovulation after this protocol and exhibit excellent fertility for lactating dairy cows [30]. Cows were artificially inseminated with semen from one of three high-fertility bulls. The use of a large number of cows and three bulls was to ensure an adequate number of excellent quality embryos produced from a single sire. Cows were evaluated using ultrasonography to determine where ovulation occurred, and seven days post-insemination, the ipsilateral horn(s) underwent a non-surgical uterine horn flushing for embryo recovery as described in Hackbart et al. [31]. Embryos were then graded for embryo quality (1 = excellent or good, 2 = fair, 3 = poor, and 4 = degenerate) and embryo stage (1 = 1-cell, 2 = 2-16 cell, 3 = early morula, 4 = compacting morula, 5 = early blastocyst, 6 = blastocyst, 7 = expanded blastocyst, 8 = hatched blastocyst) according to International Embryo Transfer Society (IETS) standards [32]. For the purpose of this study, we collected blastocyst stage embryos that were expanded and with excellent quality (Figure 3). Upon collection, embryos were placed in RNALater (Ambion, TX) to preserve RNA integrity and frozen at -20°C until RNA extraction.

### In vitro maturation, fertilization, and embryo culture

Ovaries from Holstein cows were obtained from a local abattoir, and from them antral follicles (2-6 mm) were aspirated. Recovered oocytes were then washed with warm Tyrode's albumin lactate pyruvate (TALP)-Hepes and underwent maturation, fertilization, and culture as outlined in detail by Khatib et al. [33] and briefly here. For fertilization, frozen-thawed semen from one bull used in producing the in vivo embryos was prepared by centrifuging through a discontinuous Percoll gradient (45-90%) to recover motile sperm and adjusted to a final concentration of 1 × 10^6^/ml [34]. After 8 days of culture, embryos were morphologically assessed and those with an IETS grading of 7-1 (expanded blastocyst, excellent quality) were collected individually in RNALater (Ambion) and frozen at -20°C until RNA extraction (Figure 3).

### RNA extraction, amplification, and pooling

Total RNA was extracted from individual embryos using RNaqueous Micro (Ambion). Due to limitations in the amount of RNA in each embryo, linear amplification was performed using the MessageAmp II aRNA amplification kit (Ambion). Briefly, cDNA was primed by a T7 promoter tagged poly-dT primer. After the second strand synthesis, the double stranded cDNA was used as template in an in vitro transcription reaction using T7 polymerase with unlabeled NTPs. Two rounds of linear amplification provided a proper concentration of RNA for analysis. In order to minimize biological variance between the in vivo and in vitro embryo groups for the transcriptomic comparisons, five grade 7-1 embryos from each group from the same sire were used. Samples of the amplified RNA from individual embryos were then pooled (n = 5 per pool) and prepped for RNA-sequencing.

### RNA sequencing

Libraries of amplified RNA for each pool were prepared following the Illumina mRNA-Seq protocol. Sequencing libraries were created from 50 ng samples and sequenced with Illumina's HiSeq 2000 at the University of Wisconsin-Madison Biotechnology Center. Libraries were barcoded, multiplexed, and sequenced in one HiSeq 2000 lane. A 'fragment' was defined as a cDNA fragment sequenced from both ends. Approximately 60 million fragments were sequenced for both libraries and de-multiplexed.

### Mapping reads to the reference genome

Sequencing reads were mapped to the reference genome (btau4.0) using the software package Tophat (v1.2.0) [18] implemented on Galaxy [35]. The alignment was first performed in each of the samples independently. Novel splice junctions discovered by Tophat and known splice junctions from the Ensembl annotation were then combined and supplied to Tophat for a second alignment such that the junction database was the same for each sample. This mapping strategy allows a full utilization of the novel junctions identified in both samples. A maximum of two mismatches and a minimum length of 25 bp per segment were allowed. Moreover, reads that mapped equally well to more than 40 genomic locations were discarded.

### Assembly of transcripts and estimation of abundance

Cufflinks (v1.0.3) was used to assemble transcript models from RNA-Seq alignments and to estimate transcript and gene expression [36]. This program uses graph theory to find a parsimonious set of transcript models that comply with alignments. In addition, it estimates isoform and gene expression (fragments per kilobase exon per million mapped fragments, or FPKM) by optimizing a likelihood function containing transcript abundances as parameters. In our analysis, abundances of transcripts were upper-quartile normalized and also corrected for sequence bias in order to improve expression estimates [37].

### Identification of novel transcribed units

Novel transcribed units were defined as regions containing multi-exonic transcripts that were: 1) at least 1,000 bp away from known gene boundaries; 2) of length ≥ 250 bp; 3) with an average coverage ≥ 5; 4) with less than 50% of repetitive sequences; and 5) with splice junctions supported by at least two junction alignments. For external support of our novel transcripts, cow EST alignments were downloaded from the UCSC genome browser and compared to the novel transcribed units using BEDTools [38].

### Overall gene expression and GO enrichment analysis

Differential expression of annotated genes and novel transcribed units was tested using Cuffdiff, a program part of the Cufflinks package for testing differential gene expression [35]. In our study, genes with a false discovery rate (FDR) < 0.05 were considered significant. FDR is defined as the expected proportion of false positives among all significant hypotheses [39]. The Gene Ontology (GO) enrichment analysis was performed using the GOseq (v1.4.0) package [40] that is available in the R language/environment [41]. Importantly, GOseq adjusts for gene selection bias due to difference in gene lengths, which is known to affect the variance of gene expression estimates [40]. Biological pathways with a FDR < 0.01 were considered significant.

### Analysis of alternative splicing

Differential alternative splicing of genes that showed more than one isoform was tested also with Cuffdiff using the Shannon-Jensen divergence metric to measure relative abundances between transcripts [37]. A FDR < 0.05 was chosen as the significance threshold for detecting differential alternative splicing between the two treatment groups. Alternative splicing events were classified according to Wang et al. [21] using a custom perl script.

### Validation of RNA-Seq data

For alternative splicing validation, PCR was done using cDNA produced from in vivo and in vitro embryo pools. Primers were designed for two genes (*AP2B1 *and *ZDHHC16*) that had exon-skipping events (Table 5). Fragment sizes differentiated the two transcripts for each gene. Quantitative real-time PCR (qRT-PCR) was used to validate differential expression of a select number of genes. A preliminary analysis was done to establish proper housekeeping genes for normalization as described in Vandesompele et al. [42]. Briefly, this method assumes that the ratio of two internal control genes is uniform across samples, regardless of the cell type or environmental condition. The control gene producing the smallest relative stability value M (the average of the pair-wise variation when compared with the other control genes) is deemed as having the most stable expression across samples, assuming no co-regulation, and is thus chosen for normalization. The genes guanine nucleotide binding protein (G protein) gamma 5 (*GBG5*), phosphoglycolate phosphatase (*PGP*), and cytochrome b5 type B (*QOP5F6*), showed moderate and relatively constant expression across samples in the RNA-Seq data and thus were selected as candidate housekeeping genes. Of these, *GBG5 *produced the smallest relative M-value (M = 0.19) and was designated as the internal control. Four differentially expressed genes with the highest significance [myosin light chain 7-regulatory (*MYL7*), Dual specificity protein phosphatase 6 (*DUS6*), cofilin 1 (non-muscle) (*CFL1*), and UDP-Gal:betaGlcNAc beta 1,4- galactosyltransferase, polypeptide 4 (*B4GALT4*)] were chosen for validation. Expression was measured in the five individual embryo samples composing the pools, averaged, and compared to RNA-Seq results. Primers for validation were designed to cross exon-exon junctions and are shown in Table 5. The relative gene expression values were calculated using the 2^-ΔΔCt ^method [43].

**Table 5:** Primers used for the validation of gene expression and alternative splicing

Gene	Primer sequence 5'→3'	Amplicon size (bp)
*Primers for gene expression validation*		

*GBG5*	F: TCCAGCGTCGCGGCTATGAA	94
	R: TCAAATCTGCAGCTGCCTGGGA	

*PGP*	F: TGAAGCGGCTGGGCTTCCCTAT	131
	R: GGGCACCAACATGGACAACCGA	

*QOP5F6*	F: TGGTGACGTGCACCCGAATGA	112
	R: AGCACCCACGATGGGGAAGATC	

*CFL1*	F: TGGTGTCGACGACTTACGCACT	132
	R: AGCGCCTCTCGTCTTGTAGGCT	

*B4GALT4*	F: TGATACCGGCCTCTGTGCACCT	113
	R: TGCCCTTCTGTGTCTCCACACCT	

*MYL7*	F: ACCCCAGTGGTAAAGGCGTGGT	115
	R: TCCATGGGTGTCAGGGCGAACA	

*DUS6*	F: GGGCGAACTCGGCTTGGAACTT	118
	R: AACGAGAATACGGGCGGCGA	

*Primers for alternative splicing validation*		

*AP2B1-F1*	GCCTAGATAGTCTGCTTGGCA	134
*AP2B1-R*	ACCACTGCTGACCACAGC	

*AP2B1-F2*	GCCTAGATAGTCTGGTGGGA	92
*AP2B1-R*	ACCACTGCTGACCACAGC	

*ZDHHC16-F1*	TGCTGCCATCGAGACTTA	97
*ZDHHC16-R*	GCACAGGAACCAGAGGTAGAC	

*ZDHHC16-F2*	CGACAAGAACAAACTACAGG	118
*ZDHHC16-R*	GCACAGGAACCAGAGGTAGAC	

## Competing interests

The authors declare that they have no competing interests.

## Authors' contributions

AD performed experiments and wrote the manuscript. FP and WH analyzed the data. KA participated in writing the manuscript. KH and MW collected in vivo embryos. AD and HK designed the study. All authors read and approved the final manuscript.

## Supplementary Material

Additional file 1 **The most significant biological GO pathways detected (FDR < 0.01) for differentially expressed genes**. Pathways are ranked in order of decreasing statistical significance. Click here for file
