# Supplementary material for: RNA-Seq analysis uncovers transcriptomic variations between morphologically similar in vivo- and in vitro-derived bovine blastocysts
Source: BMC Genomics. 2012 Mar 28;13:118. doi: 10.1186/1471-2164-13-118 (PMC3368723; doi:10.1186/1471-2164-13-118)
Supplement: Additional file 1 — The most significant biological GO pathways detected (FDR < 0.01) for differentially expressed genes. Pathways are ranked in order of decreasing statistical significance. [file 1471-2164-13-118-S1.DOC]

**Additional File 1** Twenty-three pathways significantly enriched (FDR < 0.01) for differentially expressed genes. Pathways are ranked in order of decreasing statistical significance

| GO ID | Ontology | Term |
| --- | --- | --- |
| 0006695 | BP | Cholesterol biosynthetic process |
| 0048731 | BP | System development |
| 0016126 | BP | Sterol biosynthetic process |
| 0030154 | BP | Cell differentiation |
| 0048513 | BP | Organ development |
| 0072358 | BP | Cardiovascular system development |
| 0072359 | BP | Circulatory system development |
| 0048869 | BP | Cellular developmental process |
| 0007275 | BP | Multicellular organismal development |
| 0048856 | BP | Anatomical structure development |
| 0005575 | CC | Cellular component |
| 0030855 | BP | Epithelial cell differentiation |
| 0032502 | BP | Developmental process |
| 0032501 | BP | Multicellular organismal process |
| 0001568 | BP | Blood vessel development |
| 0008283 | BP | Cell proliferation |
| 0009888 | BP | Tissue development |
| 0001071 | MF | Nucleic acid binding transcription factor activity |
| 0003700 | MF | Sequence-specific DNA binding transcription factor activity |
| 0050896 | BP | Response to stimulus |
| 0003674 | MF | Molecular function |
| 0001944 | BP | Vasculature development |
| 0008610 | BP | Lipid biosynthetic process |

BP: Biological process; CC: Cellular component; MF: Molecular function
